# Rapid degeneration of iPSC-derived motor neurons lacking *Gdap1* engages a mitochondrial-sustained innate immune response

**DOI:** 10.1038/s41420-023-01531-w

**Published:** 2023-07-01

**Authors:** Marian León, Javier Prieto, María Micaela Molina-Navarro, Francisco García-García, Manuela Barneo-Muñoz, Xavier Ponsoda, Rosana Sáez, Francesc Palau, Joaquín Dopazo, Juan Carlos Izpisua Belmonte, Josema Torres

**Affiliations:** 1 Departamento Biología Celular, Biología Funcional y Antropología Física, Universitat de València, Burjassot, 46100 València, Spain; 2 Gene Expression Laboratory, Salk Institute for Biological Studies, La Jolla, CA 92037 USA; 3 Unidad de Bioinformática y Bioestadística, Centro de Investigación Príncipe Felipe, 46012 València, Spain; 4 Unitat Predepartamental de Medicina, Universidad Jaume I, Castellón de la Plana, Castellón Spain; 5 Institut de Recerca and Hospital San Joan de Déu, 08950 Barcelona, Spain; 6 CIBER de Enfermedades Raras (CIBERER), ISCIII, Madrid, Spain; 7 Computational Medicine Platform, Andalusian Public Foundation Progress and Health-FPS, 41013 Sevilla, Spain; 8 Institute of Biomedicine of Seville, IBiS, University Hospital Virgen del Rocío/CSIC/University of Seville, Seville, Spain; 9 Instituto de Investigación Sanitaria (INCLIVA), 46010 València, Spain; 10 Present Address: Altos Labs, 5510 Morehouse Drive, San Diego, CA 92121 USA

**Keywords:** Cell death in the nervous system, Mitophagy, Energy metabolism

## Abstract

Charcot-Marie-Tooth disease is a chronic hereditary motor and sensory polyneuropathy targeting Schwann cells and/or motor neurons. Its multifactorial and polygenic origin portrays a complex clinical phenotype of the disease with a wide range of genetic inheritance patterns. The disease-associated gene *GDAP1* encodes for a mitochondrial outer membrane protein. Mouse and insect models with mutations in Gdap1 have reproduced several traits of the human disease. However, the precise function in the cell types affected by the disease remains unknown. Here, we use induced-pluripotent stem cells derived from a *Gdap1* knockout mouse model to better understand the molecular and cellular phenotypes of the disease caused by the loss-of-function of this gene. Gdap1-null motor neurons display a fragile cell phenotype prone to early degeneration showing (1) altered mitochondrial morphology, with an increase in the fragmentation of these organelles, (2) activation of autophagy and mitophagy, (3) abnormal metabolism, characterized by a downregulation of Hexokinase 2 and ATP5b proteins, (4) increased reactive oxygen species and elevated mitochondrial membrane potential, and (5) increased innate immune response and p38 MAP kinase activation. Our data reveals the existence of an underlying Redox-inflammatory axis fueled by altered mitochondrial metabolism in the absence of *Gdap1*. As this biochemical axis encompasses a wide variety of druggable targets, our results may have implications for developing therapies using combinatorial pharmacological approaches and improving therefore human welfare.

**Figure:**
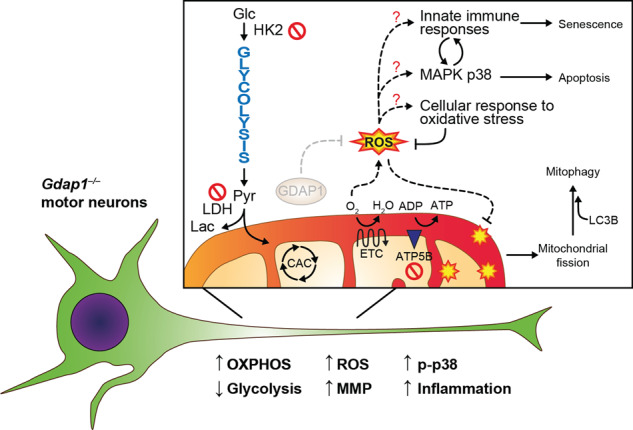
**A Redox-immune axis underlying motor neuron degeneration caused by the absence of** *Gdap1*. Our results show that *Gdap1*^*–/–*^ motor neurons have a fragile cellular phenotype that is prone to degeneration. *Gdap1*^*–/–*^ iPSCs differentiated into motor neurons showed an altered metabolic state: decreased glycolysis and increased OXPHOS. These alterations may lead to hyperpolarization of mitochondria and increased ROS levels. Excessive amounts of ROS might be the cause of increased mitophagy, p38 activation and inflammation as a cellular response to oxidative stress. The p38 MAPK pathway and the immune response may, in turn, have feedback mechanisms, leading to the induction of apoptosis and senescence, respectively. CAC, citric acid cycle; ETC, electronic transport chain; Glc, glucose; Lac, lactate; Pyr, pyruvate.

**A Redox-immune axis underlying motor neuron degeneration caused by the absence of** *Gdap1*. Our results show that *Gdap1*^*–/–*^ motor neurons have a fragile cellular phenotype that is prone to degeneration. *Gdap1*^*–/–*^ iPSCs differentiated into motor neurons showed an altered metabolic state: decreased glycolysis and increased OXPHOS. These alterations may lead to hyperpolarization of mitochondria and increased ROS levels. Excessive amounts of ROS might be the cause of increased mitophagy, p38 activation and inflammation as a cellular response to oxidative stress. The p38 MAPK pathway and the immune response may, in turn, have feedback mechanisms, leading to the induction of apoptosis and senescence, respectively. CAC, citric acid cycle; ETC, electronic transport chain; Glc, glucose; Lac, lactate; Pyr, pyruvate.

## Introduction

Charcot-Marie-Tooth (CMT) disease is the most prevalent hereditary sensory and motor neuropathy (1 in 2500). CMT disease is a clinically and genetically heterogeneous group of disorders sharing common phenotypical features, including weakness, and wasting of distal limb muscles, waned deep tendon reflexes, distal sensory loss, and skeletal deformities. Overall, the clinical traits of the disease result from progressive loss of sensory and motor nerves; affecting either the myelin shaft (CMT type 1 and 4, demyelinating) or the axon (CMT types 2-4, axonal) of motor neurons (MNs). Together, these cellular and structural defects lead to a progressive decrease in patient welfare [1–5].

Several pathological mechanisms underlying CMT2 subtypes have been described, and include defects in mitochondrial dynamics, organelle-organelle contacts, and axonal transport [6]. This pathological diversity has likely been the cause precluding CMT2 from common drug-based therapeutics [1], supporting the notion of personalized medicine as the most viable treatment strategy.

The CMT-associated gene *Ganglioside-induced Differentiation-Associated Protein 1* (*GDAP1*, MIM 606598), encodes an integral mitochondrial outer membrane protein expressed predominantly in MNs and Schwann cells [7, 8]. Similar to our *Gdap1* knockout cellular model, which renders no functional protein, previous research has defined that disease-associated missense mutations in *GDAP1* result in a premature stop codon and a shortened protein [9–14]. GDAP1 protein participates in cellular processes linked to mitochondrial function, including mitochondrial dynamics [8, 10, 15], regulation of glutathione concentration [16, 17], oxidative stress [18], mitochondrial membrane potential [17] and mitochondrial calcium buffering and defects in the mitochondria-lysosomes membrane contacts [19–21]. Although mouse [22, 23] and fly [24] models with mutations in *Gdap1* have reproduced several traits found in human CMT disease, the precise function of GDAP1 in healthy settings and its mechanistic link to CMT disease remains uncertain [25–28]. To address this, we differentiated iPSCs generated from *Gdap1*^*–/–*^ mice into MNs and found that absence of this protein results in a fragile phenotype characterized by mitochondrial dysfunction and activation of the innate immune response.

## Results

### *Gdap1*^*WT*^ and *Gdap1*^*–/–*^ iPSCs show similar potential for differentiation to mature MNs

**Fig. 1:**
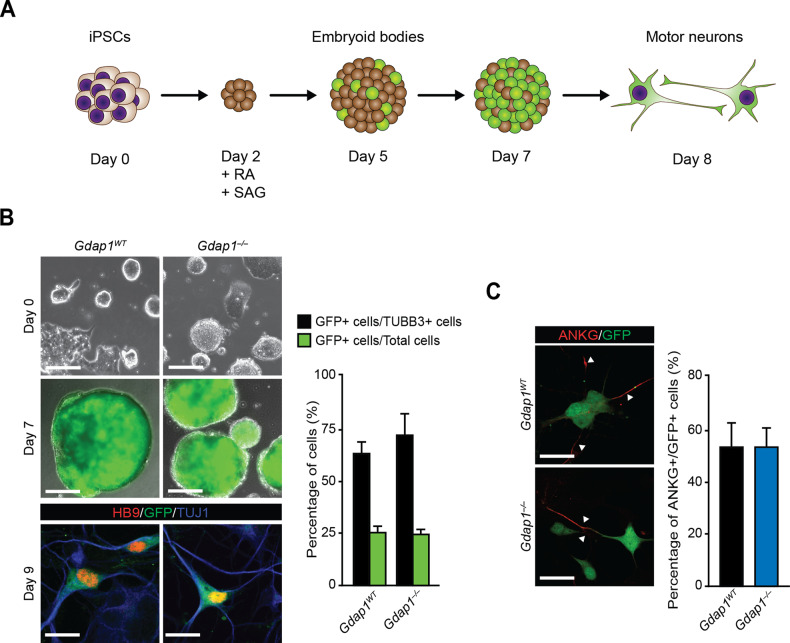
MN differentiation efficiency of *Gdap1*^*WT*^ and *Gdap1*^*–/–*^ iPSCs was similar. **A** Schematics of iPSCs MN differentiation. Retinoic acid (RA) and smoothened agonist (SAG) are indicated. **B** Upper panels, phase contrast images of undifferentiated iPSC. Middle panels, fluorescence (EGFP, green) and phase contrast merged images of EBs at d7 of differentiation. Lower panels, confocal images of MNs 2 days after plating (d9) stained with indicated antibodies. Bars, 130 μm, 500 μm, and 20 μm, respectively. Right graph, percentage of EGFP-positive cells relative to either Tbb3-positive cells (black bars) or total cells (green bars) (*n* = 3). **C** Left: confocal images of d9 MNs stained with indicated antibodies. Bars, 20 μm. Right graph, percentage of ANKG-EGFP double positive cells relative to total EGFP-positive cells. Data are represented as mean ± SEM from at least three experiments. No statistical significance between genotypes was observed (one-tailed Student’s *t*-test).

To facilitate the identification of MNs in our studies we generated reporter iPSC lines by introducing EGFP under the control of the *Mnx1* (also known as *Hb9*) promoter [29] in *Gdap1*^*WT*^ and *Gdap1*^*–/–*^ iPSCs [30]. MN differentiation was induced by retinoic acid (RA) and Smoothened agonist (SAG) stimulation for 1 week (Fig. 1A) [29, 31]. On day (d) 5, we detected EGFP expression and, on d7, embryoid bodies (EBs) were enzymatically disaggregated and cells plated in monolayer (Fig. 1B).

Differentiation efficiency was quantified by quantitative RT-PCR (qPCR), flow cytometry and immunofluorescence. On d7, EGFP fluorescent signal (Fig. 1B, middle panels) and *Mnx1* gene expression (Fig. S1A) increased similarly in both genotypes. Importantly, EGFP-positive cells displayed HB9 nuclear staining by immunofluorescence, validating the pMnx1-EGFP reporter (Fig. 1B, lower panels).

We assessed differentiation efficiency by flow cytometry at d7 and found 16% and 19% of EGFP-positive cells in *Gdap1*^*WT*^ and *Gdap1*^*–/–*^ cultures, respectively (Fig. S1B). At d9, cultures were analyzed by confocal microscopy, and the percentage of EGFP-positive cells was approximately 25% in both genotypes (Fig. 1B, green bars). At this timepoint, over 60% of EGFP-positive cells co-expressed the neuronal β-tubulin III (TUBB33) (Fig. 1B, black bars) and the neuronal polarization Ankyrin G (ANKG) markers (Fig. 1C) in both genotypes. Importantly, we detected GDAP1 protein expression in *Gdap1*^*WT*^ cultures at day 7 of differentiation while it was absent in *Gdap1*^*–/–*^ cells (Fig. S1C). Thus, as ES differentiation into MNs was not 100%, the contribution of other cell lineages to our results cannot be completely ruled out.

### *Gdap1*^*–/–*^ MNs have an abnormal cellular phenotype

**Fig. 2:**
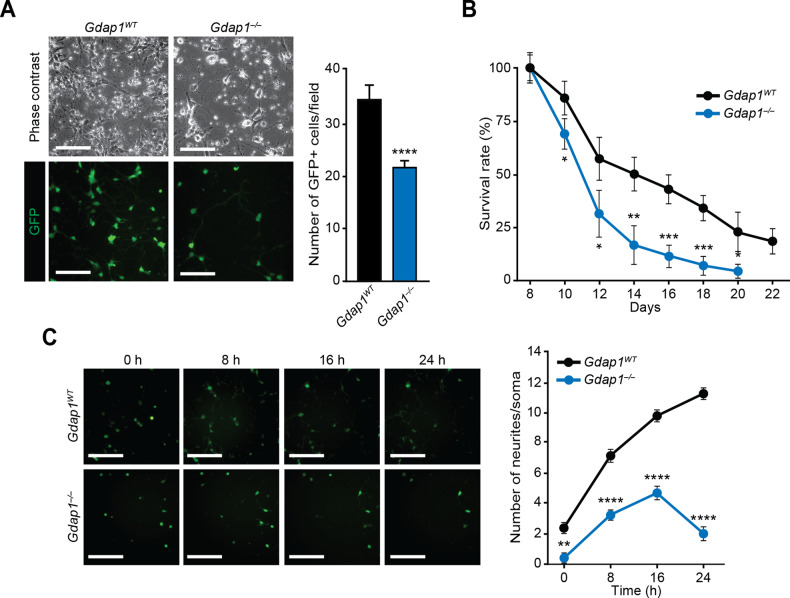
Abnormal cellular phenotype in *Gdap1*^*–/–*^ MNs. **A** Left: phase contrast (top) or fluorescence (bottom) images of differentiated cells one day after plating (d8) at the same density (1.25 × 10^5^ cells/cm^2^). Bars, 120 μm. Right: Bar diagram showing average of EGFP-positive cells per field (10 fields per well; *n* = 6 wells per group). **B** Graph showing the survival curve of EGFP-positive cells seeded as in **A**. **C** Left: INCell fluorescence images (EGFP, green) at the indicated times after seeding (d7 of differentiation). Bars, 180 μm. Right: number of neurites per soma in EGFP-positive cells at the indicated times after seeding. Data are represented as mean ± SEM from at least three experiments. Statistical significance between genotypes was assessed with the one-tailed Student’s t-test (*P < 0.05; **P < 0.01; ***P < 0.001; ****P < 0.0001).

To assess MN viability, 7-day-old EBs were disaggregated and plated at identical densities (Fig. 2A). From the next day onwards, EGFP-positive cells were manually counted for 2 weeks and compared to d8 (100%). While the percentage of EGFP-positive cells decreased over time in both genotypes, the rate of reduction in Gdap1^*–/–*^ cultures was increased, with no EGFP-positive cells beyond d20 (Fig. 2A, B). To evaluate possible paracrine signaling defects on survival, twice as many *Gdap1*^*–/–*^ cells were seeded relative to controls (Fig. S2A, B), however this did not change the viability curve. Neurite development of MNs increased rapidly following plating in wild-type controls (Fig. 2C). In contrast, while neurite development initially increased in *Gdap1*^*–/–*^ MNs, the emergence of neuronal projections stopped at 16 h post-plating and sharply decreased at 24 h (Fig. 2C).

### *Gdap1*^*–/–*^ MNs show an altered mitochondrial functionality

**Fig. 3:**
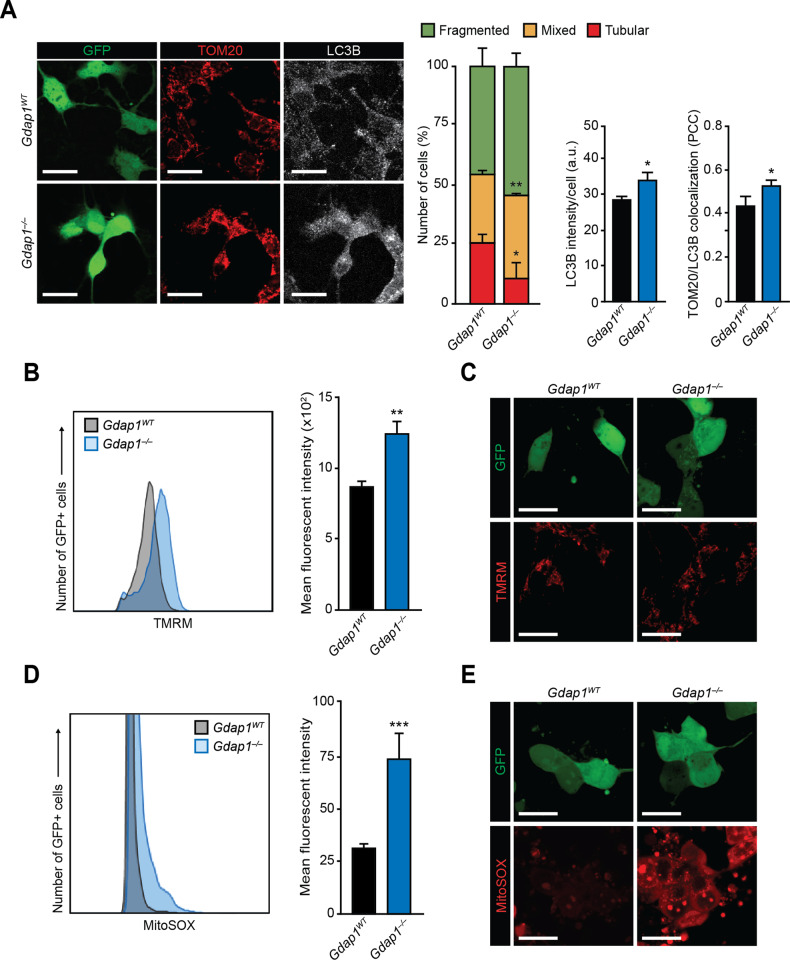
Altered mitochondrial functionality in *Gdap1*^*–/–*^ MNs. **A** Left: confocal images of MNs 2 days after plating (d9) that were fixed and stained with the indicated antibodies. Bars, 20 μm. Right: quantification of the observed mitochondrial morphologies (left graph), LC3B mean signal intensity per cell (middle graph) and colocalization of LC3B and TOM20 assessed by Pearson’s correlation coefficient (PCC) of both signals in MNs (right graph). **B** Histogram: TMRM-uptake assessed by flow cytometry of MNs at d7 of differentiation. Graph: quantification of the mean fluorescent intensities of the histograms. **C** Confocal images of MNs 2 days after seeding (d9) that were stained with TMRM. Bars, 15 μm. **D** Histogram: mitochondrial superoxide assessment by flow cytometry analysis in MNs at d7. Graph: quantification of the mean fluorescent intensities in histograms. **E** Confocal images of cultured MNs 2 days after seeding (d9) stained with MitoSOX to detect mitochondrial superoxide. Bars, 15 μm. Data are represented as mean ± SEM from at least three independent experiments. The one-tailed Student’s t-test was used to assess statistical significance between genotypes (*P < 0.05; **P < 0.01; ***P < 0.001).

As GDAP1 is a protein involved in mitochondrial dynamics [8, 32], we investigated whether this process was altered in *Gdap1*^*–/–*^ iPSC-derived MNs. Mitochondrial morphology (fragmented, tubular, or mixed) in EGFP-positive MN somas was assessed by microscopy using TOM20 as a marker for these organelles (Fig. 3A). MNs of both genotypes displayed mostly fragmented mitochondria. However, *Gdap1*^*–/–*^ cultures displayed a reduction in cells with tubular mitochondrial morphology, and an increase in those with mixed or fragmented mitochondria (Fig. 3A, middle panels and left graph). The changes in mitochondrial morphology were associated with increased autophagy and mitophagy, assessed by autophagosome LC3B staining, and Pearson Correlation Coefficient (PCC) of TOM20 and LC3B colocalization, respectively (Fig. 3A, right panels, and middle and right graphs). Along these lines, LC3B-II / LC3B-I ratios, assessed by western blot, were increased in differentiated GDAP1-null cells relative to wild-type controls (Fig. S3A). However, no significant differences were observed between genotypes in either mitochondrial mass, measured by immunoblotting for TOM20 (Fig. S3A), or in levels of the machinery governing mitochondrial dynamics in these organelles (Fig. S3B), in agreement with our previous observations in somatic and pluripotent stem cells [30, 33].

We next measured mitochondrial membrane potential (MMP) and mitochondrial superoxide anion, using TMRM and MitoSOX fluorescent probes, respectively. *Gdap1*^*–/–*^ MNs showed a significant increase of both signals at d7, measured by flow cytometry, and at d8, determined by confocal microscopy (Fig. 3B–E).

### *Gdap1*^*–/–*^ cells differentiated into MNs display an altered metabolic profile

**Fig. 4:**
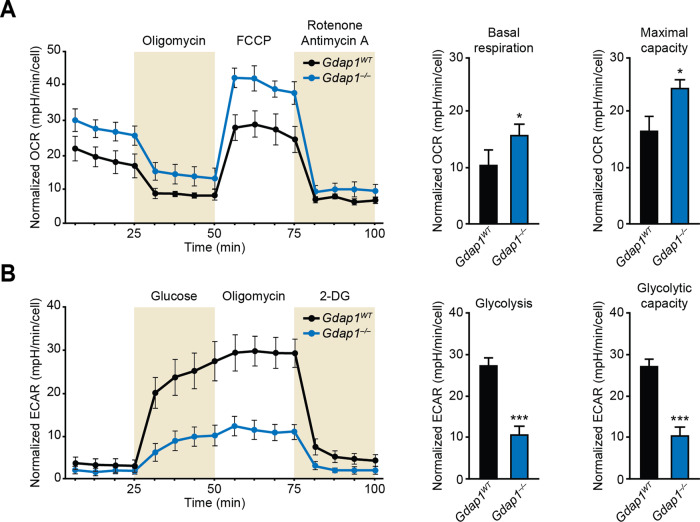
Increased OXPHOS and decreased glycolysis in *Gdap1*^*–/–*^ cells differentiated into MNs. **A** Left: oxygen consumption rates (OCR) in differentiated cultures 1 day after plating (d8 of differentiation) (*n* = 6). Right: graphs showing indicated OCR parameter values. **B** Left: extracellular acidification rates (ECAR) in differentiated cultures 1 day after plating (d8 of differentiation). Right: graphs show the values for the indicated ECAR parameters. Data are represented as mean ± SEM from at least six independent experiments. The one-tailed Student’s t-test was used to assess statistical significance between genotypes (*P < 0.05; ***P < 0.001).

Oxygen consumption (OCR) and extracellular acidification (ECAR) rates were measured as proxies of oxidative phosphorylation (OXPHOS) and glycolysis, respectively. Data analysis showed higher OCR in *Gdap1*^*–/–*^ cells compared to controls (Fig. 4A), while glycolysis and glycolytic capacity were considerably lower in *Gdap1*^*–/–*^ cultures (Fig. 4B).

**Fig. 5:**
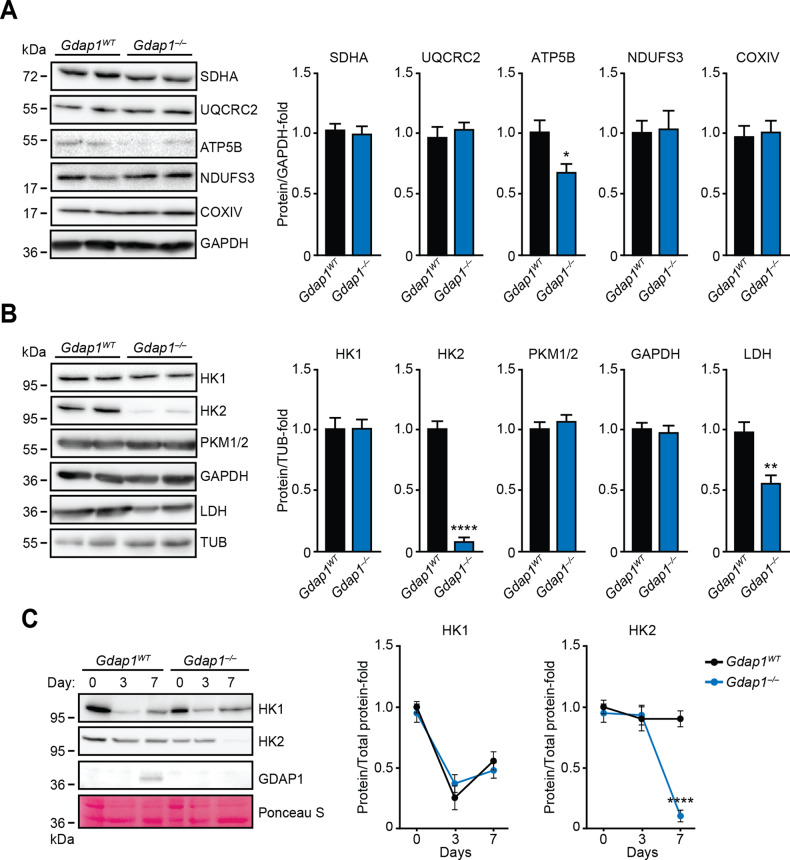
*Gdap1*^*–/–*^ cells differentiated into MNs showed altered metabolic enzyme expression. **A**, **B** Left: cell lysates of EBs at d7 of differentiation were analyzed by immunoblotting using indicated antibodies. Right: graphs showing quantification of immunoblots. **C** Left: cell lysates from undifferentiated iPSCs (day 0), or EBs at the indicated days of differentiation were analyzed by immunoblotting using the antibodies shown. Loading control: Ponceau-S staining. Right: quantification of immunoblots (*n* = 3). Data are represented as mean ± SEM from at least three independent experiments. The one-tailed Student’s t-test was used to assess statistical significance between genotypes (*P < 0.05; **P < 0.01; ****P < 0.0001).

To further investigate these metabolic differences, the major metabolic enzymes involved in OXPHOS or glycolysis were analyzed by immunoblotting. Atp5b subunit (complex V), Hk2 and Ldh enzyme expression decreased in *Gdap1*^*–/–*^ cultures (Fig. 5A, B). Interestingly, changes in hexokinase genes may participate in development of some CMT subtypes [34–36]. To rule out the possibility that HK2 downregulation was an initial genetic defect in *Gdap1*^*–/–*^ iPSCs, expression of HK1, HK2 and GDAP1 proteins was measured in undifferentiated iPSCs or during their differentiation into MNs by immunoblotting (Fig. 5C). While kinetics of HK1 expression was similar in both genotypes, expression of HK2 readily declined at d7 of differentiation in *Gdap1*^*–/–*^ cells (Fig. 5C), when GDAP1 expression is first detected (Fig. S1C).

### *Gdap1*^*–/–*^ cells differentiated into MNs show an increase of innate immune response markers and activated p38 MAPK

**Fig. 6:**
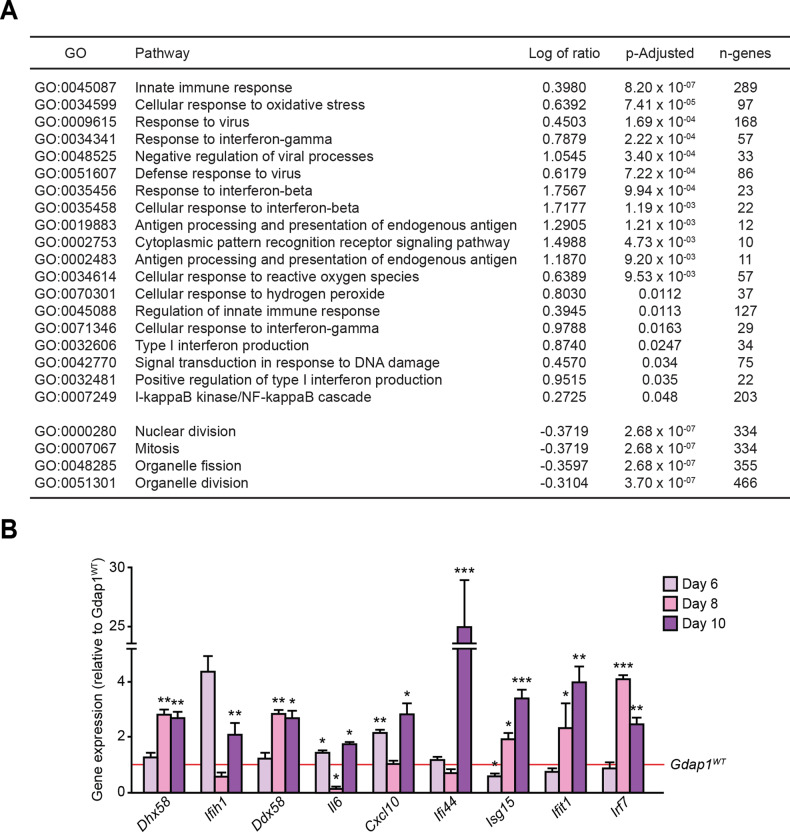
Innate immune response activation in *Gdap1*^*–/–*^ iPSCs differentiated into MNs. **A** Gene ontology analysis (GO) comparing *Gdap1*^*WT*^ and *Gdap1*^*–/–*^ MEFs gene expression profiles. Activated (top, log positive ratio) or inactivated (bottom, log negative ratio) pathways are shown. **B** iPSCs were set up to differentiate into MNs and relative gene expression was evaluated at the indicated times: d6 (EBs), d8 (1 day after plating), d10 (3 days after plating). Data are represented as the mean ± SEM from at least three independent experiments. The one-tailed Student’s t-test was used to assess statistical significance between genotypes (*P < 0.05; **P < 0.01; ***P < 0.001).

Gene ontology analysis of our previously published transcriptome data [30] revealed that *Gdap1*^*–/–*^ MEFs displayed marked upregulation of genes associated with innate immune response (Fig. 6A). In agreement with the observed oxidative stress increase in *Gdap1*^*–/–*^ MNs (Fig. 3B–E), the analysis also showed a drastic activation of markers associated with the cellular response against ROS (Fig. 6A).

Next, gene expression of these markers was analyzed during MN differentiation (Fig. 6B). Gdap1-null cells displayed an upregulation of factors induced by the activation of the innate immune response both at the EB stage (d6 of differentiation; *Il6*, and *Cxcl10*) and after seeding, at d8 (*Dhx58*, *Ddx58*, *Isg15*, *Ifit1* and *Irf7*) and d10 (*Dxd58*, *Ifih1*, *Ddx58*, *Il6*, *Cxcl10*, *Ifi44*, *Isg15*, *Ifit1* and *Irf7*).

**Fig. 7:**
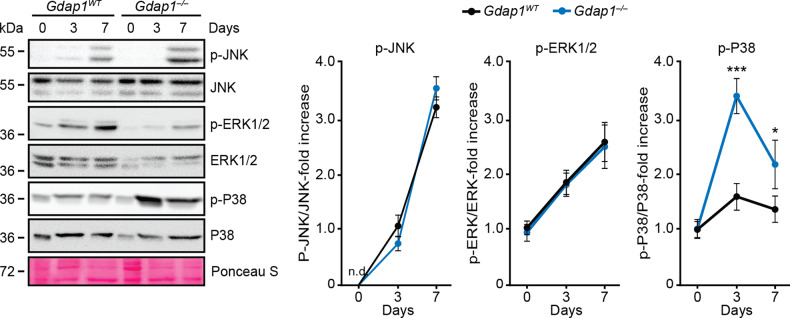
MAPK activation profile during WT and *Gdap1*^-/-^ MN differentiation. Left: cell lysates from undifferentiated or MN-differentiated iPSCs were analyzed by immunoblotting at the indicated timepoints. Loading control: Ponceau-S staining. Right: graphs showing quantification of MAPK phosphorylation (*n* = 3). Data are represented as the mean ± SEM from at least three independent experiments. The one-tailed Student’s t-test was used to assess statistical significance between genotypes (*P < 0.05; ***P < 0.001).

A relationship between ROS, innate immune response and MAPK activation does exist [37–39]. Activation of JNK1/2 and ERK1/2 MAPKs was observed in both genotypes during early differentiation (Fig. 7). In wild-type controls, p38α MAPK underwent a mild activation (around 1.5-fold) during differentiation. However, phosphorylation of this MAPK increased by more than 3-fold in Gdap1-null cultures at d3. At d7, the increased activation of p38α remained evident in GDAP1-null relative to control cultures (Fig. 7, right-most graph).

## Discussion

In this study, we found that lack of *Gdap1* induces a fragile phenotype in MNs characterized by mitochondrial dysfunction and activation of the innate immune response. Our results provide insight into Gdap1 function in health and disease.

GDAP1 is a protein of the outer mitochondrial membrane described as an accessory protein promoting fission of these organelles [8, 28, 32, 40, 41]. Conversely, our studies show that GDAP1 favors mitochondrial fusion as its absence increased the percentage of MNs with fragmented mitochondria. Further, silencing of *GDAP1* mRNA in SH-SY5Y neuroblastoma cells can induce mitochondrial fragmentation [19] and *Gdap1* deficiency in murine models has mild [21, 23] or almost no effect [22] on mitochondrial size.

Mitochondria are main sources of cellular ROS production [42], and uncontrolled production of ROS can cause fatal damage to cells [43]. By joining functional and dysfunctional mitochondria, cells distribute the contents of these organelles, maintaining homeostasis [44]. Cellular stress can lead to overwhelming mitochondrial dysfunction eventually triggering its degradation by mitophagy [45, 46], and mitochondrial fission is necessary for this process [46–49]. Our data show a correlation between increased ROS levels, mitochondrial fragmentation, and mitophagy activation in GDAP1-null MNs, suggesting a loss of mitochondrial functionality due to a rise in oxidative stress.

Defective OXPHOS can generate ROS [50]. *Gdap1*^*–/–*^ cell cultures showed a decrease in glycolysis associated with HK2 and LDH downregulation, and a rise in oxidative metabolism. While overexpression of GDAP1 induced increased MMP [17], *LDH* silencing can activate OXPHOS in several tumor cell lines [51]. Our results showing the presence of hyperpolarized mitochondria in *Gdap1*^*–/–*^ MNs suggests that mitochondria may be unable to consume MMP, possibly due to ATP5B protein downregulation. This altered metabolic profile of *Gdap1*^*–/–*^ cells correlates with a dysfunctional mitochondrial state that is likely a key factor in GDAP1 deficiency-associated cellular defects in CMT disease.

Oxidative stress has been proposed as a crucial element in various neurological disorders [52]. GDAP1 contains characteristic GST domains and, in agreement with our data, overexpression of GDAP1 stabilized glutathione concentration, reduced superoxide anion production and provided resistance to cell death induced by oxidative stress in HT22 cells [17]. Furthermore, another study demonstrated that the absence of *Gdap1* increased oxidative stress in mice [23]. In this regard, dominant mutations in *GDAP1* (associated with CMT2K) usually fall on the GST domains of the protein [53] and it has been suggested that these mutations are responsible for ROS overproduction [18].

Neuronal cell death is a hallmark of neurodegenerative diseases [54]. Here we show that *Gdap1*^*–/–*^ MNs have a fragile phenotype prone to degeneration associated with activation of the p38α MAPK, a key player in apoptotic cell death [55]. In fact, it has been suggested that persistent activation of p38 and JNK MAPKs may participate in neuronal apoptosis in neurodegenerative pathologies [56–58], including CMT2B [59].

Also, it has been observed that chronic neuroinflammation is present in several neurodegenerative diseases [60–63]. Interestingly, our results show that Gdap1-null MNs exhibit activation of the innate immune response. In agreement, *Gdap1*^*–/–*^ mice presented chronic neuroinflammation in the spinal cord and sciatic nerve [64]. The chronic inflammation observed was suggested to be part of the pathophysiology of the axonal form of *GDAP1*-linked CMT variants.

HK1 and HK2 proteins localize to the mitochondrial surface by direct interaction with the voltage-dependent anion channel (VDAC). It is known that the interaction of VDAC with these hexokinases protects cells from mitochondrial ROS production [65] and suppresses the pro-apoptotic interactions between VDAC and members of the BCL family, which lead to the opening of the mitochondrial permeability transition pore and the release of mitochondrial contents to cytosol [66–69]. Indeed, each of these processes have been previously implicated in inflammasome activation to mount an innate immune response [70]. As lack of *Gdap1* leads to downregulation of *Hk2* in MNs, the possibility that GDAP1 deficiency could lead to the opening of the mitochondrial permeability transition pore thus mounting the innate immune response is a possibility that deserves further investigation, as it may provide the molecular link to MN degeneration in CMT2, opening therefore new avenues for its therapeutical intervention.

## Materials and methods

### Cell culture, differentiation assays, reagents, and plasmids

iPSC lines were cultured in the presence of hLIF as previously described [33]. Motor neuron iPSC reporters were generated by electroporation of the *pMnx1*-EFGP reporter [29]. Differentiation of iPSC lines into MNs was carried out as described [29, 31]. Different iPS cell clones of both genotypes were included in all the experiments shown in this manuscript. All iPS cell clones used in this manuscript did not show any appreciable bias in neither their growth or differentiation properties [30]. Further details are provided in supplementary information.

### Immunofluorescence and flow cytometry

Cells were fixed in 4% PFA (in PBS), permeabilized with 0.5% Triton X-100 in PBS, blocked with 3% BSA in PBS, and incubated overnight at 4 °C with indicated primary antibodies. Next day, samples were washed with PBS and incubated with secondary antibodies in the dark. After washing with PBS, cells were incubated with Hoechst 33342 (Molecular Probes, H3570) as nuclear counterstaining, washed with PBS, rinsed with water, and mounted. For analysis of mitochondrial membrane potential, cells were incubated with 10 nm TMRM (Life Technologies, T668) for 20 min at 37 °C in the dark. For the analysis of mitochondrial superoxide, cells were incubated with 5 µM MitoSOX (Thermo Fisher Scientific, M36008) for 10 min at 37 °C in the dark. Colocalization of Tom20 and Lc3b staining was evaluated by calculating the Pearson Correlation Coefficient (PCC) using the freely available JACoP plug-in (http://rsb.info.nih.gov/ij/plugins/track/jacop.html) for ImageJ analysis software, as previously described [71]. Antibodies are listed in supplementary information.

For assessing mitochondrial membrane potential and ROS levels by flow cytometry, EBs were trypsinised and resuspended in 2% FBS-PBS solution, and incubated with TMRM 10 nm (Life Technologies, T668) or 5 μM MitoSOX (Thermo Fisher Scientific, M36008) for 5 min at 37 °C in the dark. Measurements were taken with the BD FACSCanto II cytometer (BD Biosciencies) and results analyzed with FlowJo (Tree Star, Inc.). Data from at least 10,000 cell events per sample were acquired.

### Live-cell imaging

Images of MNs in culture were captured using an IN-Cell Analyzer 2000 microscope (GE Healthcare, Inc.) with a 40X objective under controlled atmosphere conditions (5% CO_2_, 37 °C). Cells were imaged every hour (24 h total). Images were analyzed using ImageJ software (National Institutes of Health) and the number of neurites per soma was quantified by direct observation of the images.

### Western blot

Cells from EBs were lysed on ice in RIPA buffer (50 mM Tris pH 7.5, 150 mM NaCl, 0.1% SDS, 1% Triton X-100, 0.5% sodium deoxycholate) supplemented with phosphatase and protease inhibitors. Cellular lysates were used for immunoblotting with indicated antibodies. Signals in membranes were detected using ECL prime (Amersham) and images automatically captured in an Alliance Mini HD9 (UVITEC, UK) system. Acquired images were analyzed with ImageJ. Antibodies used are listed in supplementary information. For LC3B immunoblots, cells were incubated with 50 µM Chloroquine (Sigma-Aldrich, C6628) for 4 h prior lysis with ice-cold RIPA buffer. Uncropped blots can be found in Supplementary materials.

### RNA isolation and RT-qPCR analysis

Total RNA was extracted using TRI reagent (Sigma-Aldrich, T9424) and cDNA synthesized using SuperScript III reverse transcriptase (ThermoFisher Scientific, 10432122). 2–3 µl of cDNA were amplified in the StepOnePlus Real-Time PCR system (Applied Biosystems) using SYBR Premix ExTaq (Takara, RR420A). Each RT-qPCR assay was performed in duplicate and repeated at least 3 times. Primers used are listed in supplementary information.

### Microarray data analysis

Microarray data has been described [30]. In-silico analysis was carried out for the Gene Ontology (GO) terms and for the Kyoto Encyclopedia of Genes and Genomes (KEGG) Pathways using a logistic regression.

### Extracellular metabolic flux analysis

Basal and uncoupled oxygen consumption rates (OCR), or extracellular acidification rates (ECAR), were measured using a Seahorse bioanalyzer (XF96) and the Mito or Glycolysis stress test kits (both from Seahorse Bioscience, Millipore), respectively, following the manufacturer recommendations. Measurements were normalized to total number of cells per well by fluorescence microscopy on a replica-seeded plate stained with Hoechst 33342. Each experiment was conducted in quadruplicate and repeated at least 3 times. Further details can be found in supplementary information.

### Statistics

Where indicated, Student’s *t*-test was used to estimate statistical significance between two categories. Relative values (percentages) were normalized using arcsine transformation before carrying out their statistical comparison. In all assays at least 3 different iPS cell clones (*n* ≥ 3) per genotype were tested. Each data point was independently repeated at least three times. These independently repeated experiments were considered as replicates for statistical analysis. In all figures, panels display representative images from 1–2 different iPS cell clones. Bar diagrams displaying numerical results were obtained from at least 3 independent iPS cell clones (*n*) from each genotype.

## Supplementary information

Supplemental data

Figure S1

Figure S2

Figure S3

Original data WB

## Data Availability

Data supporting the present study are available from the correspondence author upon reasonable request.
