# Supplementary material for: Rapid degeneration of iPSC-derived motor neurons lacking Gdap1 engages a mitochondrial-sustained innate immune response
Source: Cell Death Discov. 2023 Jul 1;9:217. doi: 10.1038/s41420-023-01531-w (PMC10314916; doi:10.1038/s41420-023-01531-w)
Supplement: Supplementary file 1 — Supplemental data [file 41420_2023_1531_MOESM1_ESM.docx]

**SUPPLEMENTARY INFORMATION**

Supplementary data accompanying the MS by Marian Leon *et al.* supporting the conclusions of the research here described. The inventory of the content included as supplementary information follows.

INVENTORY:

- Supplementary methods
- Supplementary Tables 1-3
- Supplementary figure legends for S1-3 described in the main text.

**SUPPLEMENTARY METHODS**

**Cell culture**

For generation of Mnx1-eGfp MN reporter iPSC lines, 10x10^6^ disaggregated iPSCs (10x10^6^ cells) were mixed with the linearized pHb9-eGfp and pPYCAG-IP vectors (5 µg total DNA, 10:1 ratio) in 1 ml of Opti-MEM medium (ThermoFisher Scientific, 31985070) and electroporated (0.24 kV and 500 µFd) using the Gene Pulser Xcell Electroporation Systems (Bio-Rad, 1652660, Spain). Next day, cells were subjected to a positive selection with puromycin: 1.5 µg/ml during the first two days and 1 µg/ml during the following 5 days and cultured in 0.5 µg/ml afterwards. pHB9-EGFP (Hb9 promoter) [TJ#96] was a gift from Thomas Jessell (Addgene plasmid # 16275; http://n2t.net/addgene:16275; RRID: Addgene_16275). The pPYCAG-IP vector was a gift from Prof. Austin Smith (Living Systems Institute, University of Exeter, Exeter, UK).

For MN differentiation [30,31], iPSCs were resuspended at a concentration of 1x10^5^ cells/ml in differentiation medium [1:1 mixture of DMEM-F12 (Biowest, L0090) and Neurobasal (Thermo Fisher Scientific, 21103049) supplemented with 10% knock out serum replacement (ThermoFisher Scientific), 2 mM L-glutamine (Biowest, X0550), 1X penicillin/streptomycin (Biowest, L0022), 1X non-essential amino acids (Biowest, X0557), 1 mM sodium pyruvate (Biowest, L0642), 0.1mM β-mercaptoethanol, 1mM HEPES (Gibco, 15630-056) and 0.5X N2 (ThermoFisher Scientific)] and seeded in hanging drops (20-50 µL) on Petri dishes. On day 2, the position of the plates was restored, and fresh medium supplemented with 10 µM RA (Sigma-Aldrich, R2625) and 0,5 µM SAG (Smoothened Agonist; Calbiochem, 364590-63-6) was added for suspension culture. Media was changed every other day.

For monolayer MN culture, day 7 EBs were disaggregated enzymatically with Accutase (ThermoFisher Scientific) for 5 minutes at room temperature. Cells were resuspended in fresh medium [1: 1 mixture of DMEM-F12 (Biowest, L0090) and Neurobasal (Thermo Fisher Scientific, 21103049) supplemented with 1X B27 (Gibco, 17504-044), 2 mM L-glutamine (Biowest, X0550), 1X penicillin/streptomycin (Biowest, L0022), 1X non-essential amino acids (Biowest, X0557), 1 mM sodium pyruvate (Biowest, L0642), 0.1 mM β-mercaptoethanol, 1mM HEPES (Gibco, 15630-056), 0.5X N2 (ThermoFisher Scientific), 10 ng/ml BDNF (PeproTech, 450-02), 10 ng/ml CNTF (PeproTech, 450-50) and 10 ng/ml GDNF (PeproTech, 450-44)], counted and seeded as indicated in the text. When indicated, the medium was supplemented with 10 mM Ara-C (Sigma-Aldrich C6645) to eliminate proliferating cells. Culture dishes were pre-treated with a solution of 0.5 mg/ml poly-D, L-ornithine (Sigma-Aldrich, 8638) and 1 mg/ml fibronectin (Sigma-Aldrich, F0895-1Mg).

Different iPS cell clones of both genotypes were included in all the experiments shown in this manuscript and considered as different “n” for statistical analysis. All experiments were repeated independently at least three times. These independent replications were considered as replicates for each iPS cell line to obtain an average value that was used for statistical analysis.

**Extracellular metabolic flux analysis**

For measuring OCR and ECAR parameters using the corresponding kits (see mean text), Cells (20 000-30 000 cells per well) were plated the day before the measurements on XF96 culture microplates. Next day, media was changed to unbuffered XF Base medium supplemented with 2 mM Glutamine (glycolysis stress kit), or 25 mM glucose, 1 mM Sodium Pyruvate and 2 mM Glutamine (mitochondria stress kit) and equilibrated for 1 hour at 37 ºC without a CO2 supply. For OCR assessment, oligomycin (1 μM), FCCP (1 μM) and antimycin A/rotenone (0.5 μM) were used. For ECAR assessment, glucose (10 mM), oligomycin (1 μM) and 2-deoxy-D-glucose (50 mM) were used. Measurements were taken every 5 minutes after addition of the drugs (a total of 4 readings per reagent added) and results were normalized to the total number of cells per well, evaluated by nuclear counterstaining of replica plates as stated in the mean text.

**SUPPLEMENTARY TABLES**

| **Table 1. Primary antibodies used** | | | |
| --- | --- | --- | --- |
| **Antigen** | **Species^a^** | **Supplier^b^** | **Dilution^c^** |
| ATP5B | Ms | SCBT, sc-55597 | 1:200 (WB) |
| ANKG | Ms | SCBT, sc-166602 | 1:50 (IF) |
| COIV | Rb | CST, #4850 | 1:1000 (WB) |
| DRP1 | Rb | CST, #8570 | 1:1000 (WB) |
| p‐DRP1 | Rb | CST, #3455 | 1:1000 (WB) |
| ERK1/2 | Rb | CST, #9102 | 1:1000 (WB) |
| p‐ERK1/2 | Ms | CST, #9106 | 1:2000 (WB) |
| GDAP1 | Rb | SA, HPA014266 | 1:1000 (WB) |
| GAPDH | Rb | CST, #5174 | 1:1000 (WB) |
| HB9 | Ms | DSHB, #81.5C10 | 1:25 (IF) |
| HK1 | Rb | CST, #2024 | 1:1000 (WB) |
| HK2 | Rb | CST, #2867 | 1:1000 (WB) |
| JNK | Rb | CST, #9258 | 1:1000 (WB) |
| p-JNK | Rb | CST, #4668 | 1:1000 (WB) |
| LC3B | Rb | CST, #2775 | 1:100 (IF); 1:1000 (WB) |
| LDH | Rb | CST, #3582 | 1:1000 (WB) |
| MFN2 | Rb | SA, AV42420 | 1:1000 (WB) |
| NDUFS3 | Ms | SCBT, sc-374282 | 1:200 (IF) |
| OPA1 | Ms | BD, #612606 | 1:1000 (WB) |
| p38 | Rb | CST, #9212 | 1:1000 (WB) |
| p-p38 | Rb | CST, #4511 | 1:1000 (WB) |
| PKM1/2 | Rb | CST, #3190 | 1:1000 (WB) |
| SDHA | Rb | SCBT, sc-11998 | 1:300 (WB) |
| TOM2O | Ms | SA, WH0009804M1 | 1:500 (IF); 1:500 (WB) |
| Tubulin | Ms | SCBT, sc‐32293 | 1:5000 (WB) |
| TUJ1 | Rb | Covance, MRB‐435P | 1:1000 (IF) |
| UQCRC2 | Ms | SCBT, sc-390378 | 1:200 (WB) |

**Legend**: (a) Species in which the antibody was generated: Ms (mouse), or Rb (rabbit). (b) Antibody supplier company: CST, Cell Signaling Technology; DSHB, Developmental Studies Hybridoma Bank; SA, Sigma-Aldrich; SCBT, Santa Cruz Biotechnology. (c) Dilution used: IF, immunofluorescence; WB, protein immunodetection.

| **Table 2. Secondary antibodies used** | | | | |
| --- | --- | --- | --- | --- |
| **Antigen** | **Species^a^** | **Conjugated^b^** | **Supplier^c^** | **Dilution^d^** |
| Mouse IgG | Dk | AF–488 | TFS, A-21202 | 1:1000 (IF) |
| Mouse IgG | Dk | AF–555 | TFS, A-31570 | 1:1000 (IF) |
| Mouse IgG | Dk | AF–647 | TFS, A-31571 | 1:1000 (IF) |
| Rabbit IgG | Dk | AF–488 | TFS, A-21206 | 1:1000 (IF) |
| Rabbit IgG | Dk | AF–555 | TFS, A-31572 | 1:1000 (IF) |
| Rabbit IgG | Dk | AF–647 | TFS, A-31573 | 1:1000 (IF) |
| Mouse IgG | Gt | Peroxidase | TFS, 31432 | 1:5000 (WB) |
| Rabbit IgG | Gt | Peroxidase | TFS, 31460 | 1:5000 (WB) |

**Legend**: (a) Species in which the antibody was generated: Dk (donkey), Gt (goat). (b) Fluorophore or enzyme conjugate: AF, Alexa Fluor. (c) Antibody supplier company: TFS, Thermo Fisher Scientific. (d) Dilution used: IF, immunofluorescence; WB, protein immunodetection.

| **Table 3. Sequences of the primers used in the SYBR Green assays** | | |
| --- | --- | --- |
| **Gene** | **Forward primers (5’ to 3’)** | **Reverse primers (5’ to 3’)** |
| *Cxcl10* | CGAACTTAACCACCATCTTC | TTGATGACACAAGTTCTTCC |
| *Ddx58* | TACCTAGACCACAAACTTGG | TTACAGATATGTTGCATGGC |
| *Dhx58* | GTGTACAACACCATGTTGAG | CTGGGAGTAGCAATTCTTTG |
| *Gapdh* | GCACAGTCAAGGCCGAGAAT | GCCTTCTCCATGGTGGTGAA |
| *Ifi44* | ACCAATCACATCAAACCATC | CTATCCACGTGTGTAAGTAAAG |
| *Ifih1* | CAGATACTAGGACTGACAGC | CAAATTTCTTGCATGGTTCC |
| *Ifit1* | CAGAAAACCCTGAGTACAAC | TTCTGCTTCAACATGTTCTC |
| *Il6* | TCCTTCAGAGAGATACAGAAAC | TTCTGTGACTCCAGCTTATC |
| *Irf7* | CTACCTGTTACCAACACTTG | GCCTCTATGGTTTTAGGTTG |
| *Isg15* | ATGGAGGACTTTTGGGATAG | AGAGGCAGAGCTTTTTATTG |

**SUPLEMENTARY FIGURE LEGENDS**

**Figure S1. Motor neuron differentiation efficiency of *Gdap1^WT^* and *Gdap1^–/–^* iPSCs was similar.**

(**A**) Total RNA was extracted from *Gdap1^WT^* and *Gdap1^–/–^* EBs at day 7, *Mnx1* gene expression was then evaluated by qPCR and represented as relative gene expression to day 0 in each genotype. (**B**) Dot plots on the left, EGFP expression evaluated by flow cytometry of day 7 disaggregated EBs in *Gdap1^WT^* or *Gdap1^–/–^*. Graph on the right, percentage of cells positive for EGFP corresponding to the analysis by flow cytometry. (**C**) Upper panels, lysates of day 7 EBs from *Gdap1^WT^* or *Gdap1^–/–^* cells were analyzed by immunoblotting using the indicated antibodies. Arrowhead on the left, specific *Gdap1* band; “n.s.” on the right, non-specific signal. Lower graph, quantification of the GDAP1/GAPDH ratios. Data are represented as the mean ± SEM from at least three independent experiments. The one-tailed Student's t-test was used to compare the categories with each other.

**Figure S2. Abnormal cellular phenotype in *Gdap1^–/–^* motor neurons.**

(**A**) Left panels, representative phase contrast (upper panels) or fluorescence (lower panels) images of differentiated *Gdap1^WT^* or *Gdap1^–/–^* cells one day after plating (day 8) at different density (Gdap1^WT^, 1.25 x10^5^ cells/cm^2^; Gdap1^–/–^, 2.5x10^5^ cells/cm^2^). Scale bars, 120 μm. Right graph, number of EGFP positive cells in the cultures. (**B**) Graph showing the survival rate of cultured *Gdap1^WT^* or *Gdap1^–/–^* motor neurons (GFP-positive) seeded as in (A). Data are represented as the mean ± SEM from at least six independent experiments. The one-tailed Student's t-test was used to compare the categories with each other (* P <0.05; ** P <0.01; *** P <0.001).

**Figure S3. Altered mitochondrial functionality in in *Gdap1^–/–^* motor neurons.**

(**A**) Upper panels, cell lysates from day 7 *Gdap1^WT^* or *Gdap1^–/–^* EBs were analyzed by immunoblotting using the indicated antibodies. Lower panels, cell lysates from day 9 *Gdap1^WT^* or *Gdap1^–/–^* differentiated cells in the absence (-) or presence (CQ) of 50 µM Chloroquine were analyzed by immunoblotting using the indicated antibodies. Lower graphs, quantification of the TOM20/GAPDH (left) or LC3B-II/LC3B-I ratios (right) as indicated. The one-tailed Student's t-test was used to compare the categories with each other (* P <0.05; *** P <0.001) from at least three independent experiments. (**B**) Upper panels, cell lysates from *Gdap1^WT^* or *Gdap1^–/–^* undifferentiated iPSCs, or EBs at day 3 or day 7 of differentiation were analyzed by immunoblotting using the indicated antibodies. Ponceau S staining was used as a loading control. Upper right and graphs at the bottom show the quantification of the signals as indicated. Data are represented as the mean ± SEM from at least three independent experiments. The one-tailed Student's t-test was used to compare the categories with each other.
