# Supplementary figures and images for: Rapid degeneration of iPSC-derived motor neurons lacking Gdap1 engages a mitochondrial-sustained innate immune response
Source: Cell Death Discov. 2023 Jul 1;9:217. doi: 10.1038/s41420-023-01531-w (PMC10314916; doi:10.1038/s41420-023-01531-w)

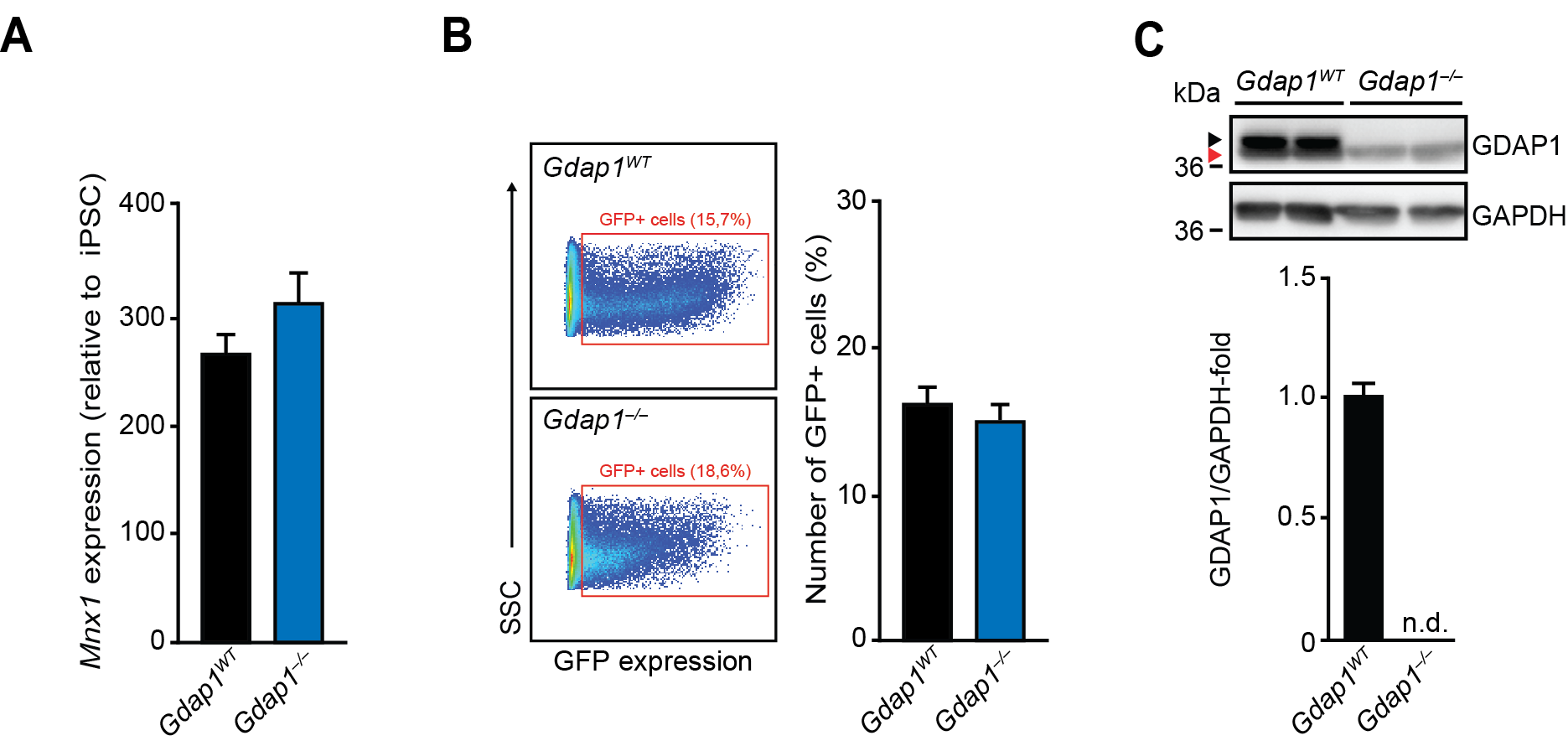

Supplement: Supplementary file 2 — Figure S1 [file 41420_2023_1531_MOESM2_ESM.png]

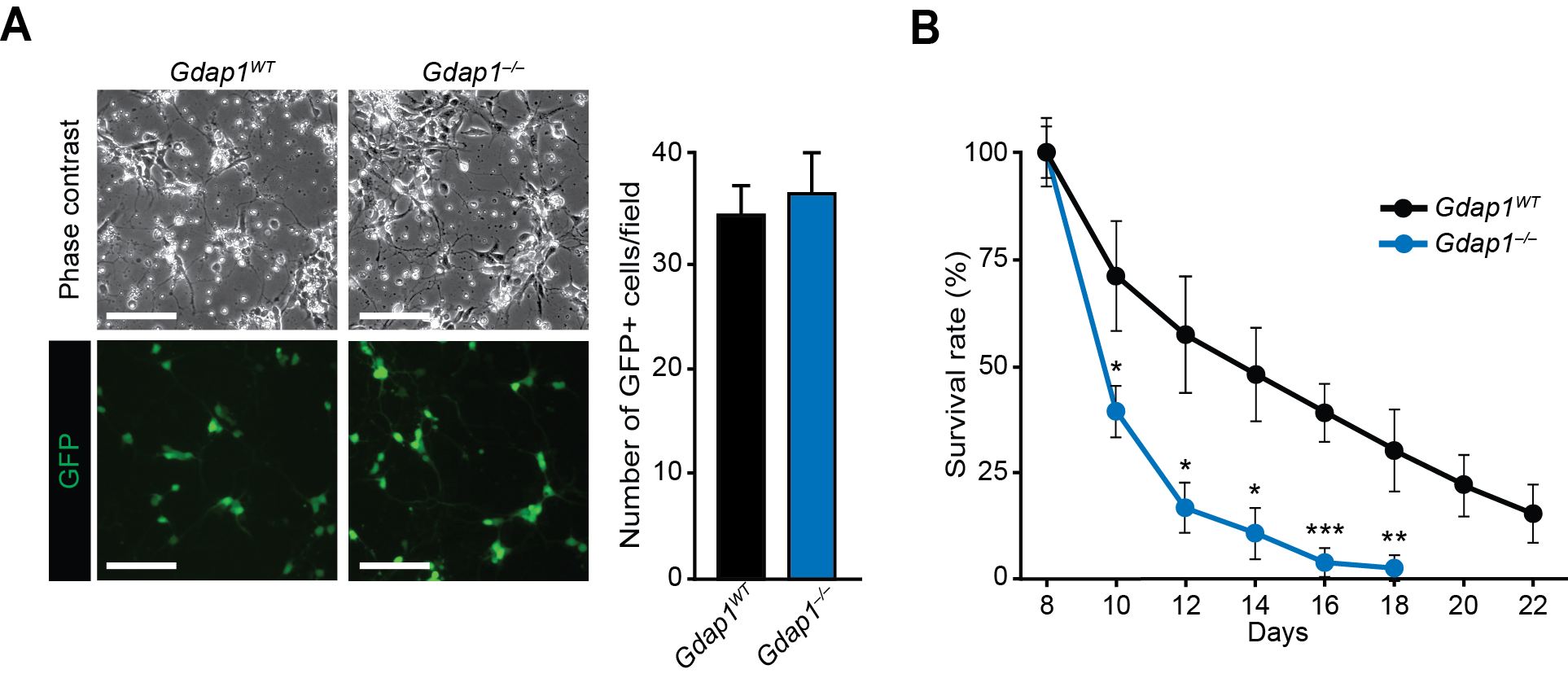

Supplement: Supplementary file 3 — Figure S2 [file 41420_2023_1531_MOESM3_ESM.png]

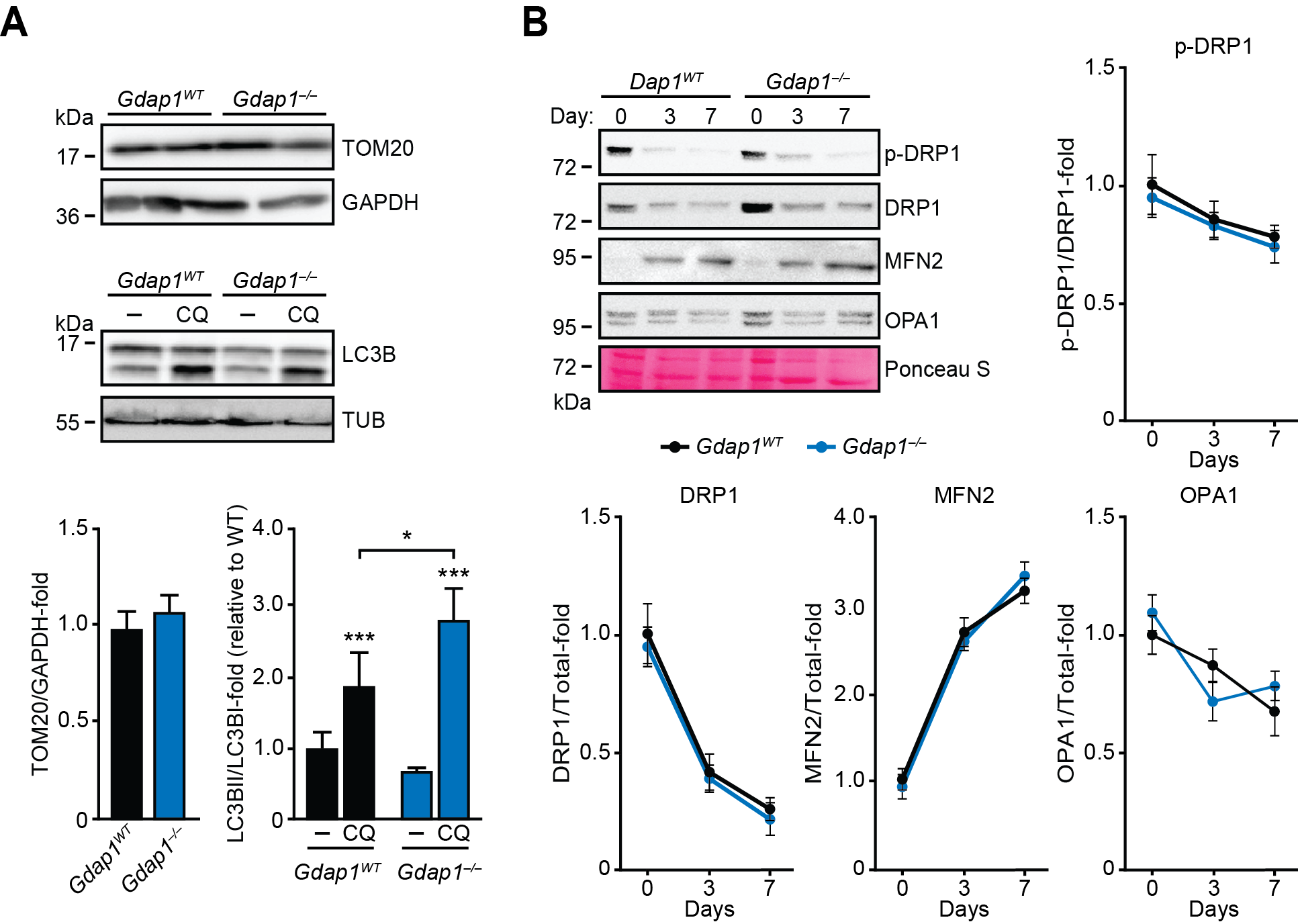

Supplement: Supplementary file 4 — Figure S3 [file 41420_2023_1531_MOESM4_ESM.png]

## Original data files-1

Figure 5A & Supp. Figures 1C, 3A

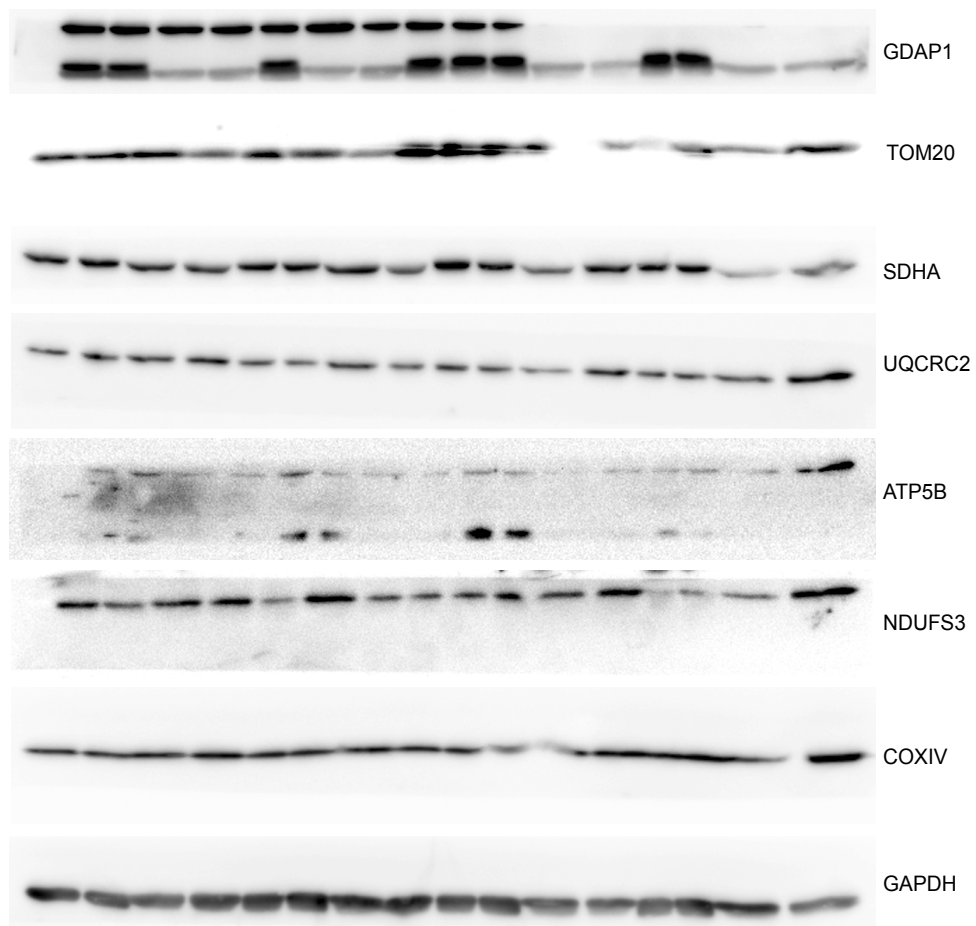

Figure 5B

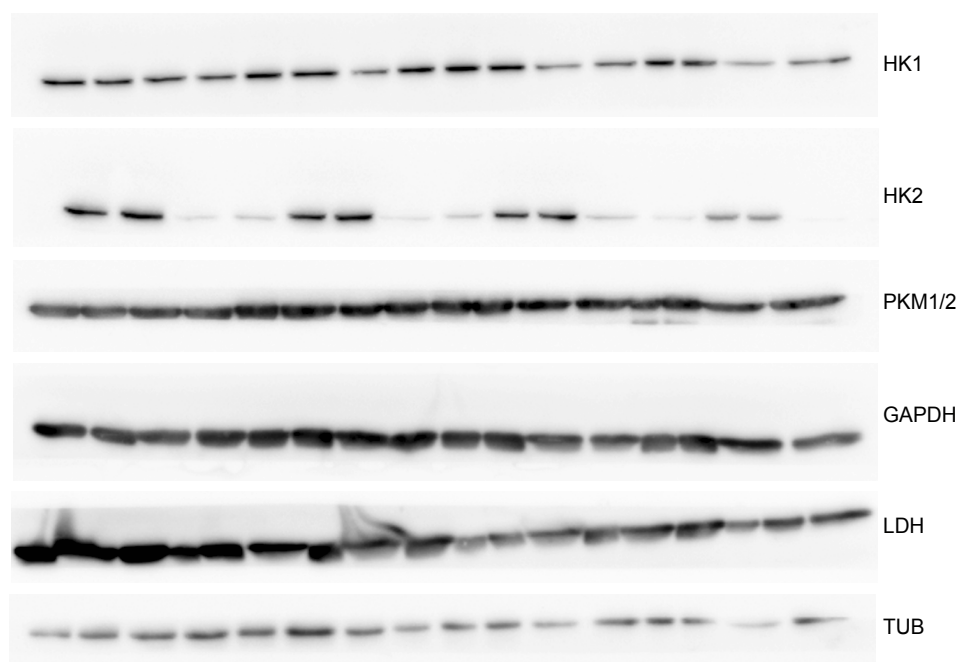

Supp. Figure 3A

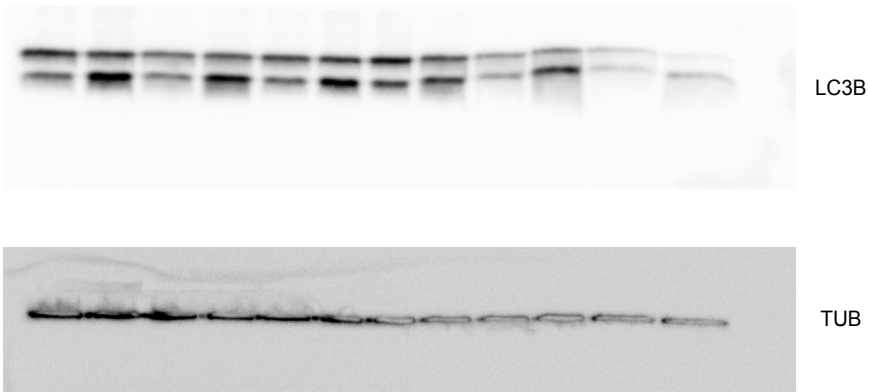

Figure 5C

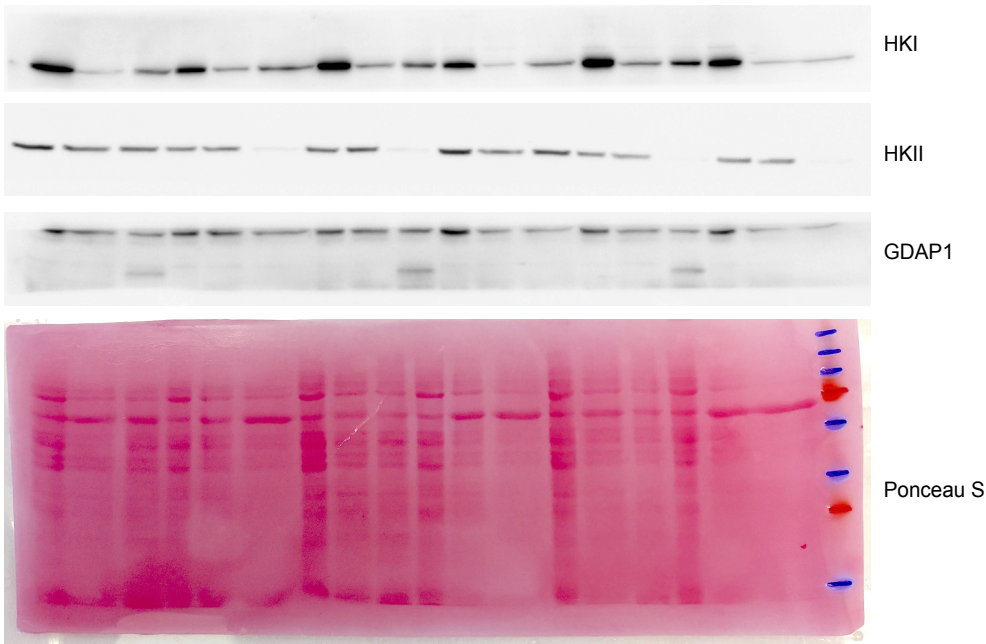

Figure 7 & Supp. Figure 3B

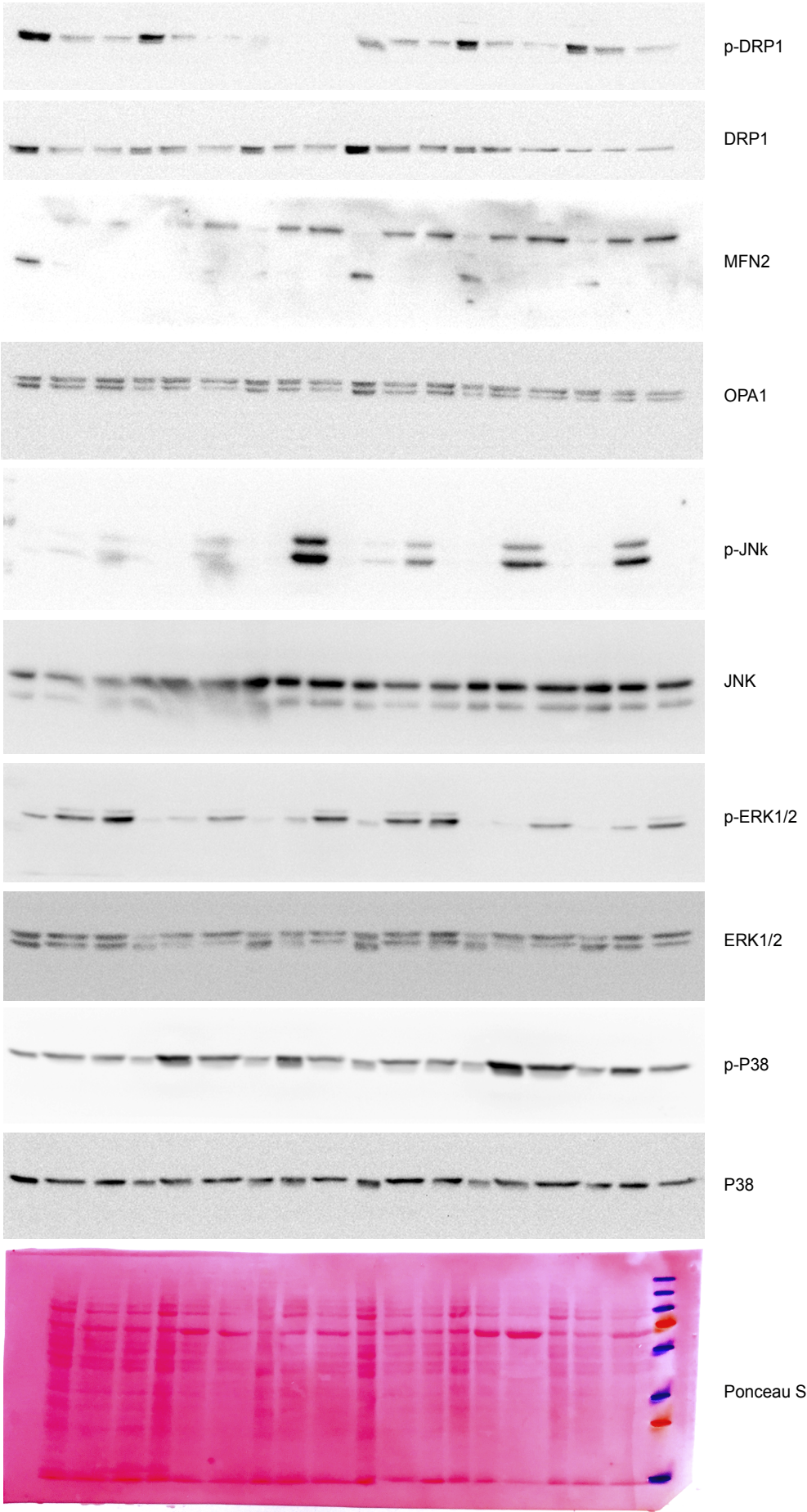

Supplement: Supplementary file 5 — Original data WB [file 41420_2023_1531_MOESM5_ESM.pdf]
